# Evaluation of an Actinomycin D/VX-680 aurora kinase inhibitor combination in p53-based cyclotherapy

**DOI:** 10.18632/oncotarget.198

**Published:** 2010-10-30

**Authors:** Bhavya Rao, Ingeborg M.M. van Leeuwen, Maureen Higgins, Johanna Campbell, Alastair M Thompson, David P Lane, Sonia Lain

**Affiliations:** ^1^ Centre for Oncology & Molecular Medicine, University of Dundee, Ninewells Hospital & Medical School, Dundee, DD1 9SY, Tayside, UK; ^2^ Department of Microbiology, Tumor and Cell biology, Karolinska Institutet, Stockholm SE-171 77, Sweden; ^3^ p53 Laboratory (A*STAR), 8A Biomedical Grove, #06-06, Immunos S138648, Singapore

**Keywords:** p53, cyclotherapy, Actinomycin D

## Abstract

p53-based cyclotherapy is proving to be a promising approach to palliate undesired effects of chemotherapy in patients with tumours carrying p53 mutations. For example, pre-treatment of cell cultures with Nutlin-3, a highly-selective inhibitor of the p53-mdm2 interaction, has been successfully used as a cytostatic agent to protect normal cells, but not p53-defective cells, from subsequent treatment with mitotic poisons or S-phase specific drugs. Here we sought to evaluate whether low doses of Actinomycin D (LDActD), a clinically-approved drug and potent p53 activator, could substitute Nutlin-3 in p53-based cyclotherapy. We found that pre-treatment with LDActD before adding the aurora kinase inhibitor VX-680 protects normal fibroblasts from polyploidy and nuclear morphology abnormalities induced by VX-680. However, and although to a lower extent than normal fibroblasts, tumour cell lines bearing p53 mutations were also protected by LDActD (but not Nutlin-3) from VX-680-induced polyploidy. We also report that a difference between the response of p53 wild-type cells and p53-defective cells to the LDActD/VX-680 sequential combination is that only the former fail to enter S-phase and therefore accumulate in G1/G0. We propose that drugs that incorporate into DNA during S-phase may perform better as second drugs than mitotic poisons in cyclotherapy approaches using LDActD as a cytostatic agent.

## INTRODUCTION

p53, also known as the “guardian of the genome” [1], is perhaps the most studied tumour suppressor to date. p53 activation occurs in response to a variety of stresses, including DNA damage, and can lead to different outcomes in cells [2]. One is a protective cytostatic effect that prevents damaged cells from multiplying until lesions have been sufficiently repaired. Alternatively, and perhaps in response to a combination of signals to p53 indicating that the damage is irreparable, p53 activity triggers programmed cell death. Highlighting its important role in cancer, the *P53* gene is mutated or deleted in about 50% of human adult solid tumours [3]. In many of the remaining tumours the p53 pathway is inactivated due to alterations in factors regulating p53 [4]. For instance, high expression of mdm2 [5], which is p53's primary negative regulator (mdm2 binding to p53 inhibits p53's function and enhances p53 degradation by the proteasome), is a frequent event in tumours expressing intact p53. Other events leading to impaired p53 function in tumours include loss of expression of the mdm2 inhibitor p14ARF [6] or the expression of viral oncogenes [7].

Most classic chemotherapeutic agents preferentially target rapidly dividing cells. However, selectivity towards cancer cells is limited and therefore toxicity to normal tissues remains a major problem in the clinic. In addition, most of these agents are highly mutagenic either by causing damage to DNA (directly or indirectly) or, as in the case of mitotic poisons, by disrupting chromosome distribution. These effects contribute to the killing of tumour cells, but also have undesired consequences on normal tissues that lead to neutropaenia, hair loss, and malaise during treatment and also to an increase in the risk of second tumours later in life. Cyclotherapy is an emerging strategy that aims at reducing the toxicity, mutagenicity, and aneuploidy in normal tissues associated with classic chemotherapy [8,9]. In this regard, the current knowledge on small-molecule agents that preferentially induce p53 mediated cell-cycle arrest rather than apoptosis could be of great utility. It is generally accepted that a mild activation of p53 should prevent the entry of normal cells into S-phase and mitosis [8,9]. Hence, using low doses of p53 activators to induce the protective cytostatic effect of p53, could protect normal cells with intact p53 from the toxicity of S- and M-phase poisons, including many of the classic cancer therapeutics. Instead, and if the p53 activating agent is sufficiently selective, tumour cells with defects in the *P53* gene would continue progressing through the cell cycle and thus remain sensitive to standard therapy. Hence, in principle, this approach should be particularly beneficial for patients with tumours that carry deletions or inactivating mutations in p53 [8].

Indeed, the use of a small molecule, Nutlin-3, a highly selective inhibitor of the p53-mdm2 interaction [10], has led to promising results. In cultured cells, this non-genotoxic p53 activator can protect wild-type p53 cells, including non-tumour cells, from the cell killing effects of DNA synthesis and mitotic poisons [11-13]. It is most encouraging that protecting normal tissues with Nutlin-3 from the effects of a mitotic poison has also proven to be a successful strategy in a preclinical model [14]. However there are two major drawbacks with Nutlin-3; first, its use in the clinic still needs to be approved and, second, it needs to be administered at high doses *in vivo* [10,14]

In the cyclotherapy study presented here, we investigate whether low doses of Actinomycin D (LDActD), a clinically-approved antineoplastic agent [15], could mimic the protective effects of Nutlin-3 on normal cells in culture. This concept was based on evidence that low nanomolar doses of ActD are not significantly genotoxic [16], effectively increase p53 levels and transcription function and induce the expression of a panel of genes that overlaps with Nutlin-3-induced genes [17]. In addition, this highly-potent compound can cause p53-dependent reversible cell-cycle arrest in normal keratinocytes [17].

Despite the similarities between LDActD and Nutlin-3 in cells with regards to their effects on the p53 pathway, their mechanisms of action differ. Whereas Nutlin-3 binds directly to mdm2, ActD inhibits RNA synthesis by binding to GC-rich regions in DNA and is especially effective at disrupting ribosomal RNA biosynthesis [18-21]. This causes a nucleolar stress that is sensed by p53 [22,23]. According to the current model, nucleolar stress caused by ActD is able to enhance the interaction between mdm2 and ribosomal proteins, such as L11, resulting in the impairment of mdm2-dependent degradation of p53 [24]. These mechanistic considerations led us to test whether LDActD could indeed substitute Nutlin-3 in a p53-based cyclotherapy approach.

## RESULTS

### Differences in the p53 response after treatment with Nutlin-3 and Actinomycin D

**Figure 1:**
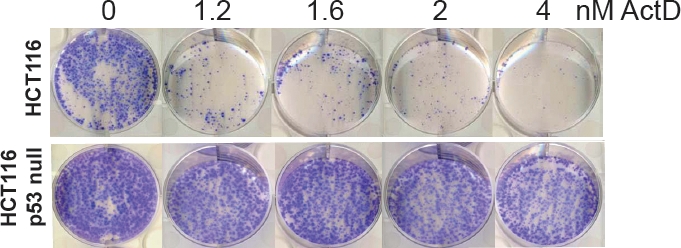
LDActD's effects on cell survival are p53-dependent HCT116 cells with wild-type p53 or knockout for p53 were treated with ActD for 72 hours, after which they were fixed and stained with Giemsa.

In spite of their different mechanisms of action, the ability of both LDActD and Nutlin-3 [11] to decrease cell growth is highly dependent on wild-type p53. This was clearly shown in a recent publication [17] and corroborated here for LDActD with a colony-growth assay comparing HCT116 colon carcinoma cells expressing wild-type p53 with HCT116 cells lacking p53 (Figure 1).

**Figure 2:**
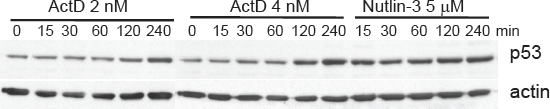
Effects of ActD and nutlin-3 on p53 levels MCF7 cells were treated with 2 nM ActD, 4 nM ActD or 5 μM Nutlin-3 for the indicated time points. Whole cell extracts were prepared and p53 was detected by Western Blotting using the DO1 antibody. Actin was detected as a loading control.

According to the existing literature, there are no clear differences between the effects of Nutlin-3 and LDActD on the p53 pathway. Phosphorylation of p53 on serine 15 is not substantially increased in response to exposure to either, whereas phosphorylation of p53 on serine 46 is increased to a similar extent by both agents [17]. Phosphorylation of p53 at serine 392 was initially observed after LDActD treatment, but not following Nutlin-3 treatment [17]. A more detailed analysis showed that phosphorylation at this site is also detectable in response to Nutlin-3 [25]. However, because of the nature of their p53-activation mechanisms, some differences in the speed at which p53 is induced by Nutlin-3 and ActD could be expected. As shown in Figure 2, Nutlin-3 treatment quickly increases p53 levels, its performance being much faster than ActD's in the same experiment. According to current understanding, ActD's effect on mdm2 requires that nucleoli are disrupted and that there are sufficient molecules of ribosomal proteins to inhibit mdm2. This is perhaps a much longer process than directly inhibiting mdm2 in order to stabilise p53. Whether this difference in the speed at which the p53 protein is increased is also partly due to differences in the permeability and/or stability of the compounds cannot yet be ruled out. Alternatively, Nutlin-3 and ActD may increase p53 in certain cell subpopulations only, i.e. in cells at particular stages of the cell cycle. These considerations may have repercussion for the use of ActD in a cyclotherapy setting.

### Actinomycin D protects normal cells from VX-680-induced aneuploidy

It has been recently shown that pretreatment with Nutin-3 protects normal keratinocytes from polyploidy caused by the small molecule aurora kinase inhibitor VX-680 (or MK-0457) [11]. Building upon the experience with the Nutlin-3/VX-680 combination, we carried out similar experiments using normal human dermal fibroblasts (NHDFs) pretreated with LDActD, instead of Nutlin-3, and subsequently exposed to VX-680. VX-680 is a potent aurora kinase inhibitor tested for the treatment of solid tumours and leukemia. Clinical trials were stopped due to electrocardiograph QTc interval prolongation in one patient. Nevertheless, because cyclotherapy studies using Nutlin-3 have been performed in combination with VX-680, we proceeded with the LDActD/VX-680 combination as this facilitates comparisons between Nutlin-3 and LDActD as potential cytostatic agents in p53-based cyclotherapy.

**Figure 3:**
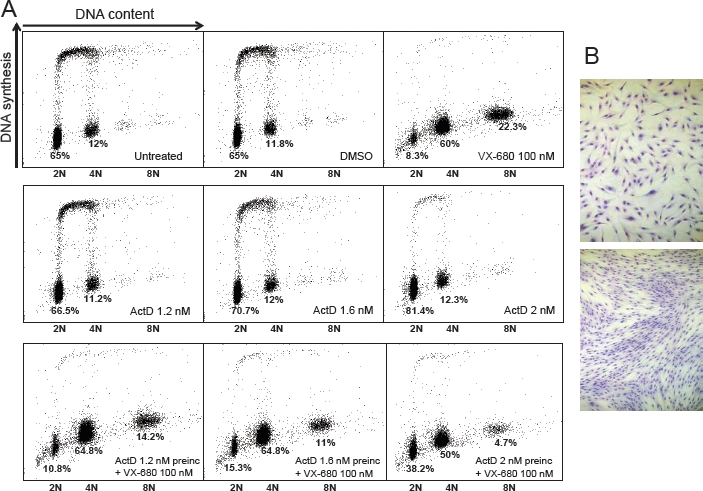
Protection of normal human dermal fibroblasts from VX-680-induced polyploidy (A) Pretreatment with low dose ActD decreases the proportion of 8N cells caused by VX-680 in NHDF cells. ActD was added 24 hours post-seeding. VX-680 was added 48 hours post-seeding. Cells were harvested 96 hours after seeding. (B) NHDFs were treated with 4 nM ActD for 72 hours, fixed and stained with Giemsa (top panel). In the bottom panel, ActD was removed from the medium and cells were allowed to proliferate for 6 days before Giemsa staining.

Activity of aurora kinases peaks during the G2 and M phases of the cell cycle and, accordingly, VX-680's killing effect is mainly by induction of endoreduplication in the absence of mitosis resulting in polyploidy and, eventually, cell death by mitotic catastrophe [26]. Normal dividing cells are susceptible to this effect and indeed proliferating NHDFs respond to VX-680 growth inhibition (Figure 1 supplemental data). Within 48 hours of treatment there was a significant increase in the number of polyploid 8N cells in cultures treated with 100-500 nM VX-680 (Figure 3A and Figure 2 supplemental data). At the higher doses of VX-680, the proportion of 8N cells diminished in association with an increase in the number of sub-diploid population. In order to ensure on-target effects of the compound we chose to work at a concentration of VX-680 of 100 nM.

At 1.2 and 1.6 nM, ActD had a weak cell-cycle inhibitory effect on NHDF cells that became more evident with a slightly higher (2 nM) concentration (Figure 3A). The number of dead cells with a sub-diploid DNA content was only marginally increased by these doses of ActD. Interestingly, NHDF cells are able to resume proliferation upon removal of ActD (Figure 3B).

Preincubation of the NHDF cells with 1.2, 1.6 and 2 nM ActD before VX-680 treatment markedly diminished the proportion of polyploid 8N cells (and to some extent the number of sub-diploid cells) in a dose-dependent manner, demonstrating that LDActD's cytostatic effect can protect normal cells from the effects of aurora kinase inhibition. A marked difference in the number of cells in G1 was also observed between VX-680 treated (8.3% of cells in G1) and ActD/VX-680 treated cells (up to 38.2% of cells in G1) (Figure 3A). This suggests that at least part of the protection of NHDFs by LDActD from VX-680 is due to inhibition of the G1/S transition by LDActD. The importance of this point is highlighted in the Discussion section.

**Figure 4:**
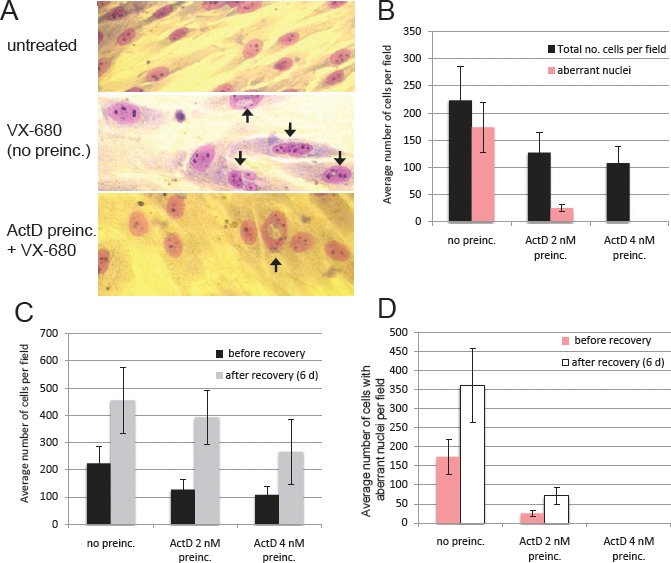
Protection of normal human dermal fibroblasts from VX-680-induced nuclear abnormalities (A and B) NHDF cells were treated for 48 hours with VX-680 alone (labelled as no preinc.), or treated for 48 hours with VX-680 after preincubating with the indicated amounts of ActD for 24 hours. Thereafter cells were fixed, stained Giemsa (A) and counted (B). Arrows in (A) indicate cells with abnormal nuclei. (C and D) NHDF cells treated as in A and B were allowed to recover for 6 days in drug-free medium. The growth of the cultures in drug-free medium is quantified in panel (C) by counting the total number of cells per field before and after the recovery period. The average number of cells per field with aberrant nuclei before and after the recovery period is shown in panel (D). Error bars correspond to standard deviations.

We also quantified the number of NHDF cells with aberrant nuclear morphologies induced by VX-680 (Figure 4). In the cultures treated with VX-680 only, a large proportion of the cells have abnormal nuclei. However, this number was markedly lower in the cultures that had been preincubated with LDActD (Figures 4A and 4B). Moreover, NHDF cells recovered from the ActD/VX-680 combination and proliferated after removal of the compounds (Figure 4C). In these recovery experiments, the number of cells with aberrant nuclei in the cultures treated with VX-680 alone as well as with the LDActD/VX-680 combination did rise after the recovery period. However, the number of abnormal cells remained comparatively low in the cultures recovering from exposure to the LDActD/VX-680-combination regimen (Figure 4D).

**Figure 5:**
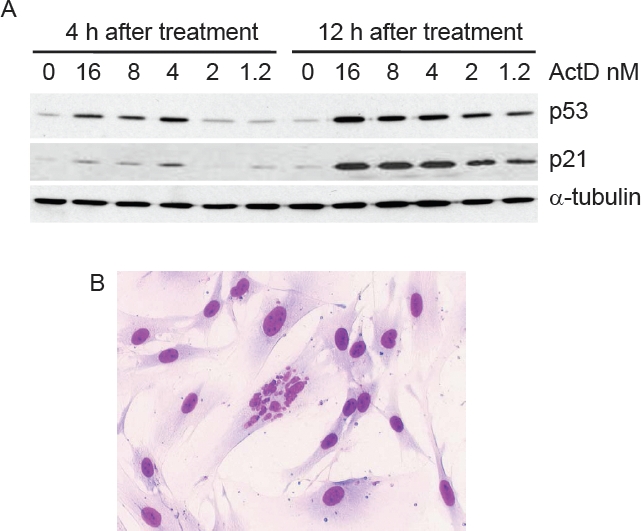
Induction of p53 by ActD in normal fibroblasts (A) NHDF cells were treated with the indicated amounts of ActD for 4 or 12 hours. p53 and p21 were analysed by Western blotting. α-tubulin was detected as a loading control. (B) Example of nuclear abnormalities in NHDF cells expressing a dominant negative form of p53. The percentage of cells with this sort of aberrant nuclei was 2.5 fold higher than in the corresponding cells with intact p53. Severely damaged cells, such as the one in the center of this picture, only appeared in the fibroblasts with dominant negative p53.

Finally, Figure 5A shows that in NHDFs, LDActD clearly increased the levels of p53. As expected from the results in Figure 2, long-term exposure to low doses of ActD is required to increase p53 levels. The levels of the p53 downstream transcriptional target p21, a well-established cell-cycle arresting protein were also increased by LDActD. This suggests that the protective effect of LDActD in NHDF cells could be due to the activation of p53 by this compound. We also observed that cells with abnormal nuclei are more frequent in NHDF cells expressing a dominant negative form of p53 than in the corresponding control NHDFs (Figure 5B).

### Response of p53 defective tumour cell lines to the ActD/VX-680 combination

**Figure 6:**
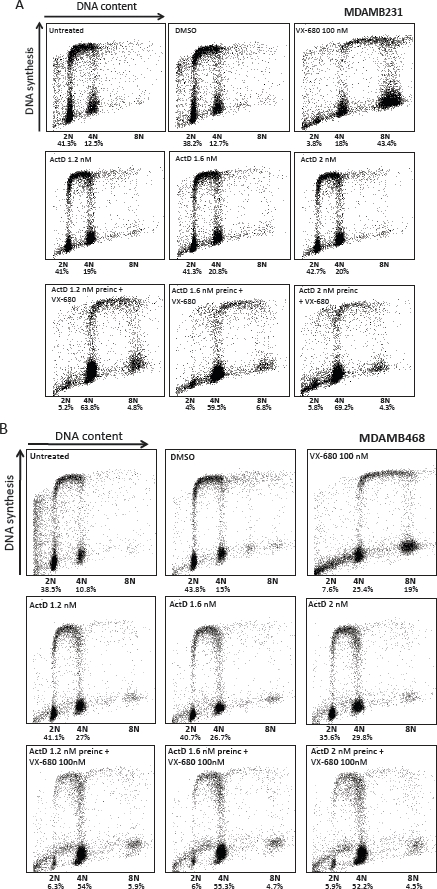
Effect of the LDActD/VX-680 combination on p53-mutant tumour cells Cell-cycle distribution analyses of MDAMB231 (A) and MDAMB468 (B) cells treated with the indicated drugs as described in Figure 3A. The proportion of 2N, 4N and 8N cells not synthesising DNA (i.e. not incorporating BrdU) is given.

**Figure 7:**
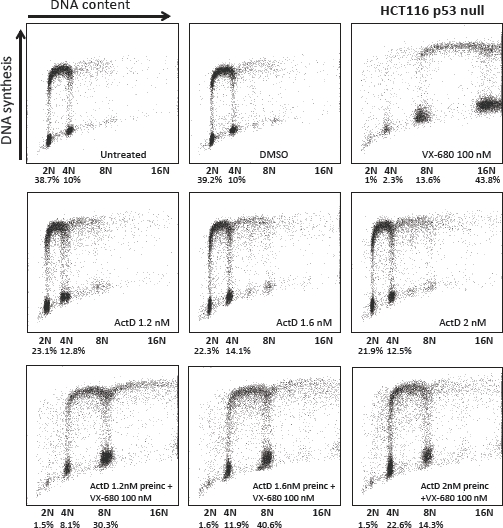
Effect of the LDActD/VX-680 combination on p53-null tumour cells Cell-cycle distribution analysis of HCT116 p53-null cells treated with the indicated drugs as described in Figure 3A. The proportion of 2N, 4N and 8N cells not synthesising DNA (i.e. not incorporating BrdU) is given.

**Figure 8:**
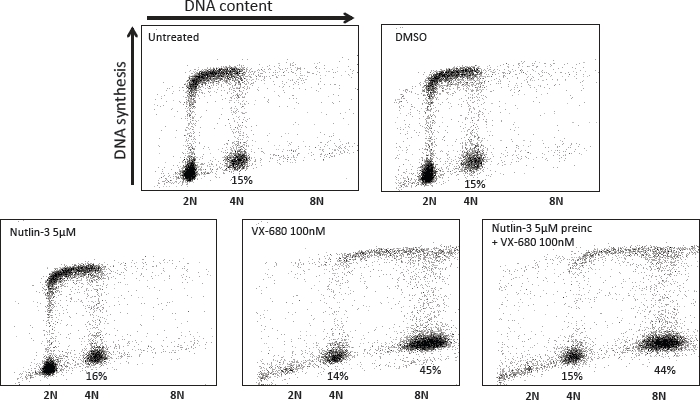
Effect of the Nutlin-3/VX-680 combination on p53-mutant tumour cells MDAMB231 cells were treated sequentially with Nutlin-3 and VX-680 as described in Figure 6A for the ActD/VX-680 combination. Cell-cycle distribution analysis was carried out by FACS 72 hours after Nutlin-3 addition.

The next question in this study was whether preincubation with LDActD affected the outcome of VX-680 treatment in tumour cells with defects in the *P53* gene. For this analysis we chose to work with two human breast cancer cell lines carrying p53 mutations frequent in cancers. These cell lines, MDAMB231 and MDAMB468 express transcriptionally inactive p53 with mutations R280K and R273H, respectively. In both cell lines, as in normal fibroblasts, VX-680 treatment alone led to an increase in 8N-polyploid cells (Figure 6). However, and unexpectedly, LDActD pre-treatment at the doses required to protect normal fibroblasts also reduced the population of VX-680-induced 8N cells in these two tumour cell types. The effect of LDActD in these tumour cells differs from the effect in normal fibroblasts in that most MDAMB231 and MDAMB468 cells accumulate in a tetraploid state, whereas co-treatment of NHDF cells also leads to a rise in the proportion of cells with a 2N DNA content. In fact, it can be seen that exposure to LDActD on its own led to a small accumulation of cells with 4N DNA content in both MDAMB231 and MDAMB468 cultures. It may be that this small effect of LDActD becomes more apparent in cultures treated with a compound that, like VX-680, impairs the G2/M transition. A similar response to LDActD combined with VX-680 was observed in HCT116 p53-null cells (Figure 7), strongly supporting that the effect seen in mutant-p53 tumour cell lines is not dependent on the presence of mutant p53. Unlike ActD, Nutlin-3 had no effect on the cell-cycle progression of p53 mutant tumour cells either on its own or in combination with VX-680 (Figure 8). This is in line with the high selectivity of Nutlin-3 as an inhibitor of the p53/mdm2 interaction.

**Figure 9:**
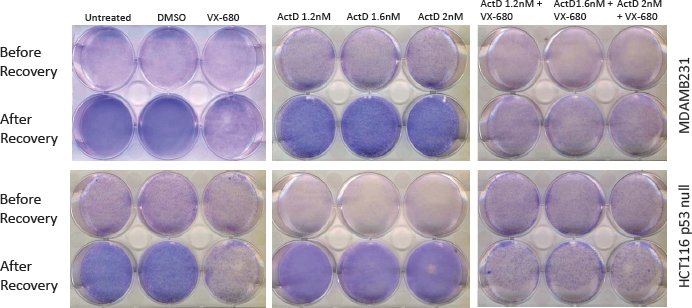
Recovery of p53 deficient cells from the LDActD/VX-680 combination Actinomycin D does not improve the rate of recovery from VX-680 treatment in tumour cells with MDAMB231 cells with mutant p53 (top plates) nor in HCT116 p53-null cells (bottom plates). Cells were treated as described in Figure 3A, fixed and stained with Giemsa. In the top panels, drugs were removed from the medium and cells were allowed to proliferate for 8 days in fresh medium.

However, despite the ability of LDActD to weaken VX-680-induced polyploidy in cells with defective p53, when cells are allowed to recover from treatment upon drug removal, the cell density in the LDActD/VX-680-treated and VX-680-treated cultures is similar after the recovery period (Figure 9).

In conclusion, we observed that, even at low doses, ActD has p53-independent effects, including a p53-independent G2/M arresting effect that becomes accentuated in the presence of an aurora kinase inhibitor. Therefore, we cannot exclude that LDActD could protect a proportion of tumour cells with defective p53 from the toxicity of this mitotic poison.

## DISCUSSION

**Figure 10:**
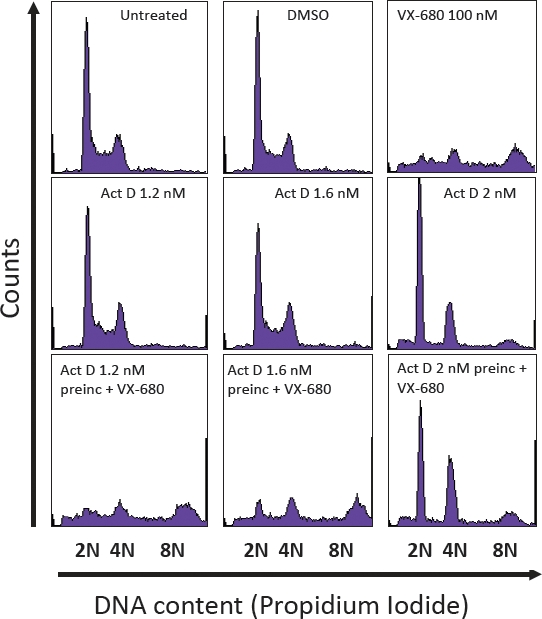
Effect of the LDActD/VX-680 combination on p53-wild type tumour cells HCT116 cells (p53 wild-type) were treated as described in Figure 7 and cell cycle distribution analysis was carried out by FACS.

Motivated by the promising results obtained by combining Nutlin-3 with mitotic poisons and the similar p53-dependent effects of LDActD and Nutlin-3, we have sought to investigate the potential of LDActD as the cytostatic agent in a p53-based cyclotherapy approach. In summary, we found that:

1. Low non-genotoxic doses of ActD have a reversible cytostatic effect on normal human dermal fibroblasts and protect them from aneuploidy induced by VX-680.
2. However, treatment with LDActD before VX-680 also prevents the appearance of 8N cells in tumour cells expressing mutant p53 or lacking p53, even though LDActD pre-treatment does not weaken the growth inhibitory effect of VX-680.
3. Unlike p53-mutant cells and HCT116 p53 knockout cells, NHDFs as well as HCT116 p53 wild-type cells (Figure 10), accumulate in G1/G0 in response to LDActD on its own or in combination with the subsequent VX-680 treatment. This suggests that wild-type p53 is responsible for reducing S-phase entry upon LDActD treatment.

The evidence presented here supports that pretreatment with a low non-genotoxic dose of ActD, a clinically-approved drug, could be an adequate approach for cyclotherapy studies in animal models, but at the same time raises two issues for consideration.

First, the therapeutic window for ActD is narrow: concentrations of ActD above 1 nM are required in order to protect normal fibroblasts effectively, but concentrations above 10 nM ActD are genotoxic [17]. Additionally, concentrations of ActD above 10 nM also affect NHDF viability (Figure 3 supplemental data). However, Nutlin-3 also shows problems in this regard. This compound is not very potent in cells (only active in the micromolar range) and needs to be administered to animals at very high doses [10,14].

Second, we have evidence suggesting that ActD could weaken, at least to some extent, the effect of aurora kinase inhibitors in tumour cells with mutant p53. Our expectation is that ActD could contribute to decrease the growth rate of p53-mutant tumours but, at the same time, prevent cell killing by mitotic poisons. Indeed, and in agreement with this, low doses of the intercalating agent Adriamycin have been reported to protect p53-null tumour cells from taxol derived mitotic poisons [27].

Nevertheless, as argued below, we believe that pretreatment with LDActD might have a future in p53-based cyclotherapy. A key difference between the effects of LDActD in p53 wild-type cells and p53-defective cells is that only the former undergo arrest in the G1/G0 phase of the cell cycle and therefore they do not enter S-phase. Hence, we envisage that clinically-approved drugs that incorporate into DNA during S-phase, such as gemcitabine and cytosine arabinoside, may constitute a better choice following pretreatment with LDActD than mitotic poisons. In support of this future direction, efficacy has been demonstrated in vitro using Nutlin-3 pretreatment and S-phase-specific chemotherapeutics as a second treatment [13]. In conclusion, LDActD merits further evaluation in the context of p53-based cyclotherapy.

## MATERIALS AND METHODS

### Cells and reagents

NHDF cells were bought from Promocell. NHDF cells expressing a dominant negative form of p53 were obtained by stable transfection with an expression vector for the murine DD-p53 dominant-negative mutant and neomycin resistance as described [28]. HCT116 and HCT116 p53-null cells were a kind gift from Bert Vogelstein (Baltimore). H1299 and MCF-7 cells were bought from ATCC. MDAMB231 and MDAMB468 cells were bought from CRUK. Actinomycin D was purchased from Sigma and stock solution was 2 mg/ml in Ethanol. VX-680 was a gift from Phil Cohen (Dundee) and stock solution was 10 mM in DMSO.

(±)-Nutlin-3, referred to as Nutlin-3 here, was purchased from Cayman Chemicals. It must be noted that this product comprises two enantiomers, active (-)-Nutlin and inactive (+)-Nutlin. Therefore, 5 μM Nutlin-3 corresponds to 2.5 μM of the active enantiomer. The stock solution was 50 mM in DMSO.

### Cell cycle analysis

Fluorescence Activated Cell Sorting (FACS) was used to analyse the cell-cycle distribution. Cells were seeded in 6-well tissue culture coated plates at required density on Day 1 and compounds were added 24 hours post-seeding at required doses on Days 2 (cytotastic agent) and 3 (VX-680). After required treatment times, Bromodexoyuridine (BrdU), supplied by Sigma, was added to cells for 30 minutes at final concentration of 30 μM. Cells were subsequently harvested using trypsinisation, spun down and resuspended in 1 ml of PBS. Cells were then fixed in 3 ml of ice-cold ethanol and stored for at least 1 hour at 4°C. Cells were pelleted and resuspended in 2 ml of warm pepsin at 1 mg/ml (Sigma) in 30 mM HCl pH1.5, for 30 minutes at 37°C with gentle shaking. Cells were then pelleted by centrifugation and resuspended in 1 ml of 2M HCl for 20 minutes at RT. Cells were washed in excess PBS before washing with the antibody buffer (0.5% w/v BSA, 0.5% v/v Tween-20 in PBS) and then resuspended in 200 μl of mouse primary antibody to BrdU (Becton Dickinson) in antibody buffer for 1 hour at RT. After washing with PBS, the samples were resuspended in 200 μl of secondary anti-mouse IgG FITC conjugated antibody (Sigma) and incubated at RT for 30 minutes in the dark. The cells were then washed with PBS and collected by centrifugation and finally resuspended in 500 μl of PBS containing 25 μg/ml propidium iodide (Invitrogen) to stain DNA. Samples were analysed using a Becton Dickinson FACScan operated by the CELLQuest software.

### Giemsa Staining and Image Capture

Cells were seeded in 6-well plates at the required density on Day 1 and treated as indicated. Cells were fixed with methanol for 7 minutes after which methanol was removed and plates left to dry. Cells were stained with 5% Giemsa for at least 1 hour before being washed with distilled water to remove excess stain. Plates were left to dry and then the morphology of the cell nuclei was assessed using a Zeiss light microscope under the 10X or 32X objective.

### Analysis of cell extracts by Western blotting

Cells grown in 6-well plates were harvested by first washing them twice in 2 ml of ice-cold PBS before being lysed using 1 x Novex NuPage LDS sample buffer (LDS), supplied by Invitrogen. All samples were then scraped into separate 1.5 ml centrifuge tubes. Cell lysates were heated to 90°C for 5 minutes, sonicated twice for 10 seconds, and centrifuged at 13200 rpm for 5 minutes. Protein concentrations were then normalised after determining the protein concentration using the BCA assay kit (Perbio). Once levelled, DTT was added to a final concentration of 0.1 M.

Proteins were separated on NuPAGE TM 4-12% Bis-Tris gels in NuPAGE TM MOPS SDS running buffer (Invitrogen) at 130V for approximately 100 minutes. Proteins were transferred to Immobilon-P membranes (Millipore) for 90 minutes at 35 V with 1 x NuPAGE TM transfer buffer (Invitrogen). Membranes were incubated at RT for 45 minutes in blocking solution (PBS/ 0.1 % v/v Tween-20/ 5% w/v non-fat dried milk) with gentle shaking. Following blocking, membranes were incubated with the appropriate primary antibody (diluted in blocking solution) for 1 hour at RT or overnight at 4°C. Membranes were washed 4 times for 5 minutes at RT in PBS/0.1 % Tween-20, before incubating with the appropriate HRP-conjugated secondary antibody (Dako) diluted in the blocking solution for 45 minutes at RT. Membranes were washed a further 5 times for 10 minutes each in PBS/0.1 % v/v Tween-20, and then a further 2 washes for 5 minutes in PBS at RT before detection of chemiluminescent signals with ECL solutions according to manufacturer's instructions (Amersham Biosciences).

## SUPPLEMENTAL FIGURES


