# Supplementary material for: Evaluation of an Actinomycin D/VX-680 aurora kinase inhibitor combination in p53-based cyclotherapy
Source: Oncotarget. 2010 Oct 30;1(7):639–50. doi: 10.18632/oncotarget.198 (PMC3248124; doi:10.18632/oncotarget.198)
Supplement: Supplementary file 1 [file oncotarget-01-639-s001.pdf]

Supplemental Data (Rao et al.,)

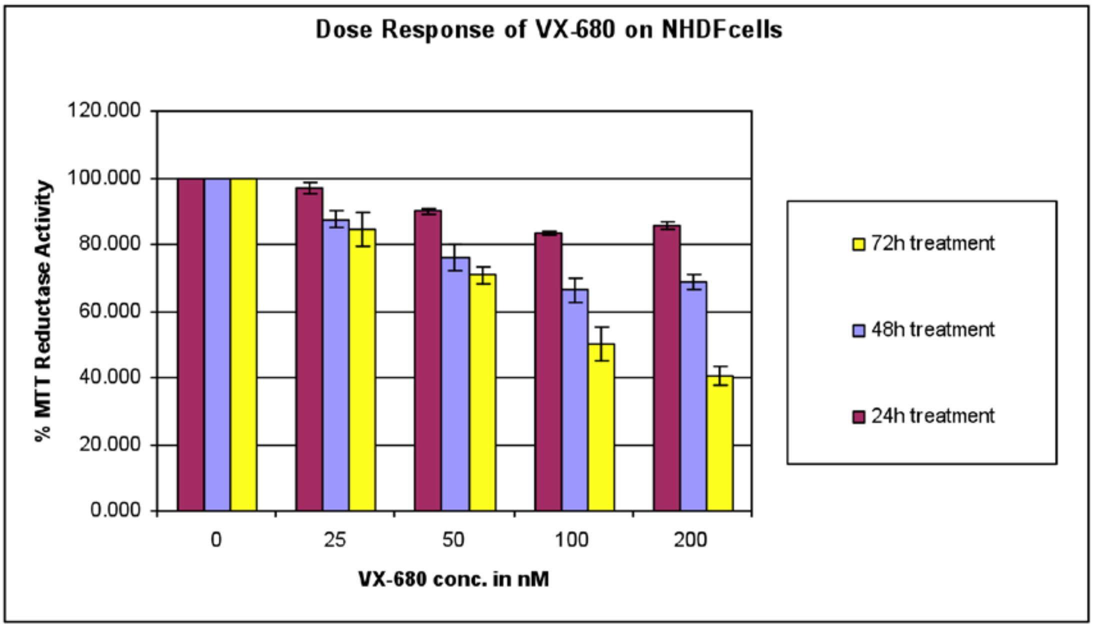

Figure S1: Cell viability of NHDF cells was assessed using the MTT assay as instructed by the manufacturer (Sigma). NHDF cells were treated with indicated doses of VX-680 for 24, 48 or 72 hours. VX-680 was added 24 hours post seeding.

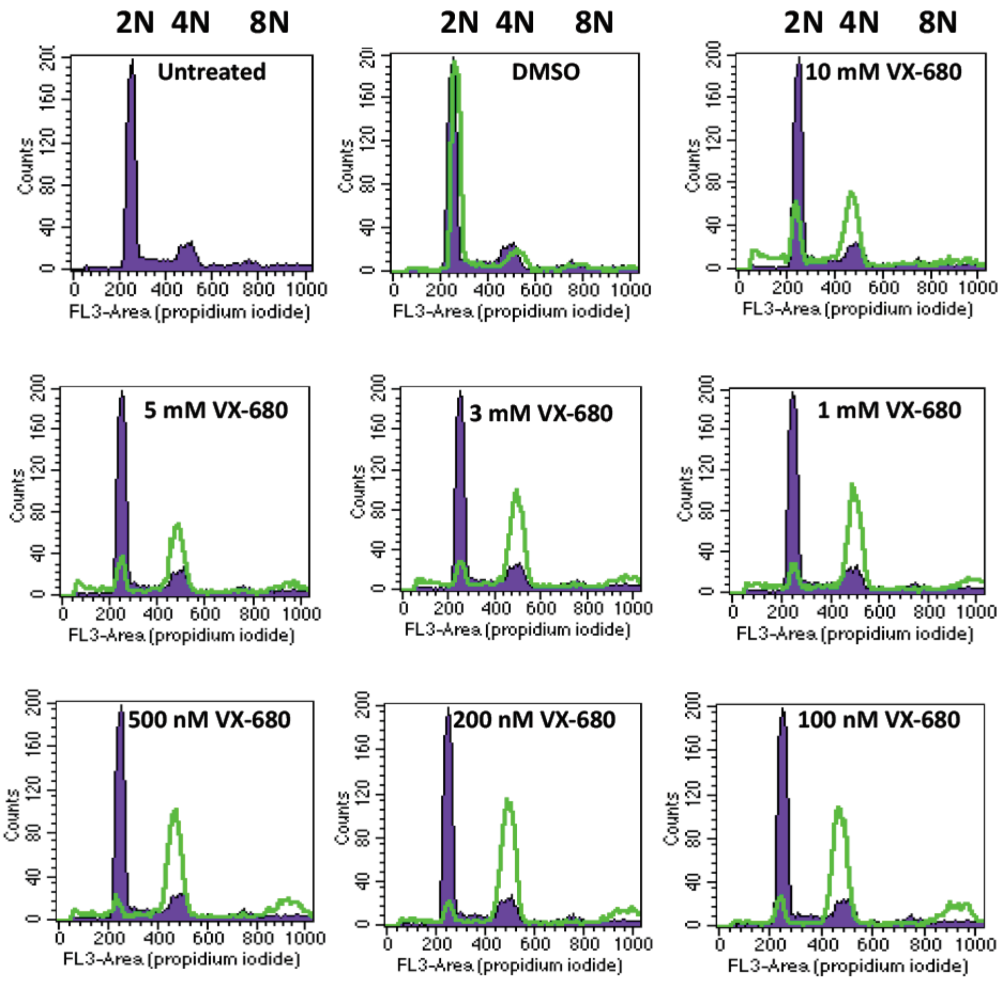

Figure S2: NHDF cells were treated with indicated doses of VX-680 24 hours post seeding. 48 hours post-addition, cells were incubated with 30µM BrdU for 30 minutes and then harvested for cell-cycle distribution analysis by FACS. The treated samples are shown here in green transparent histograms overlaid on the blue untreated profile to compare the effects of VX-680 on NHDF cells.

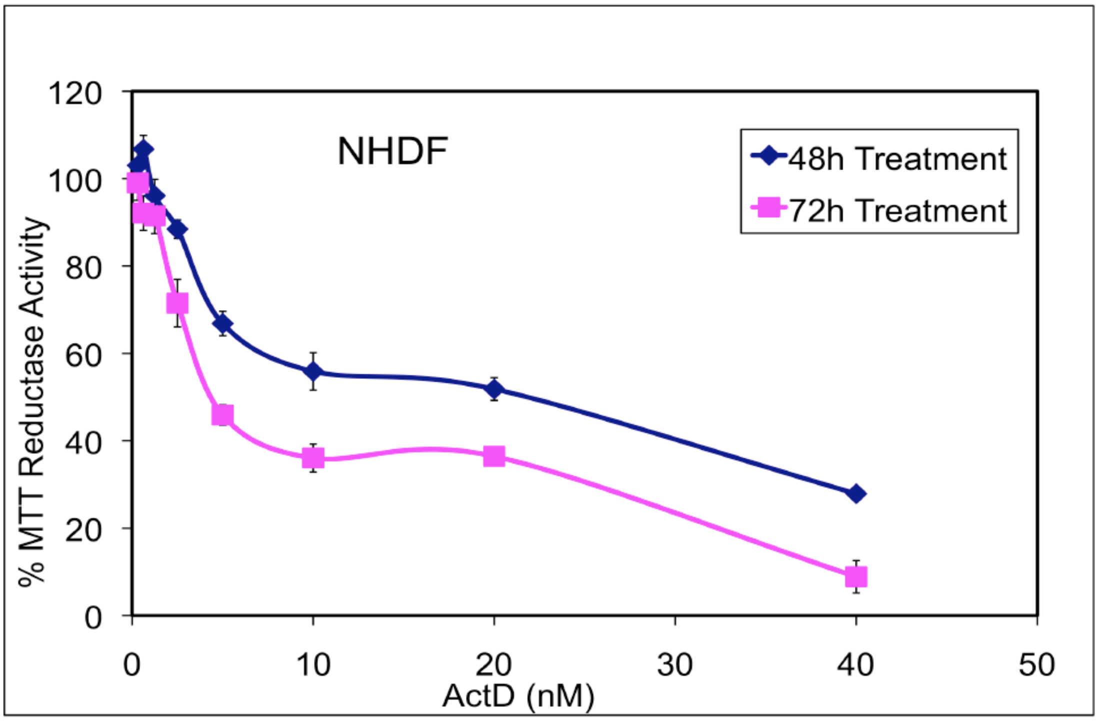

Figure S3A: Cell viability of NHDF cells was assessed using the MTT assay. NHDF cells were treated with different concentrations of ActD (40 nM to 0.3 nM subsequently diluted 1:2) for 48 or 72 hours. Act D was added 24 hours post-seeding.

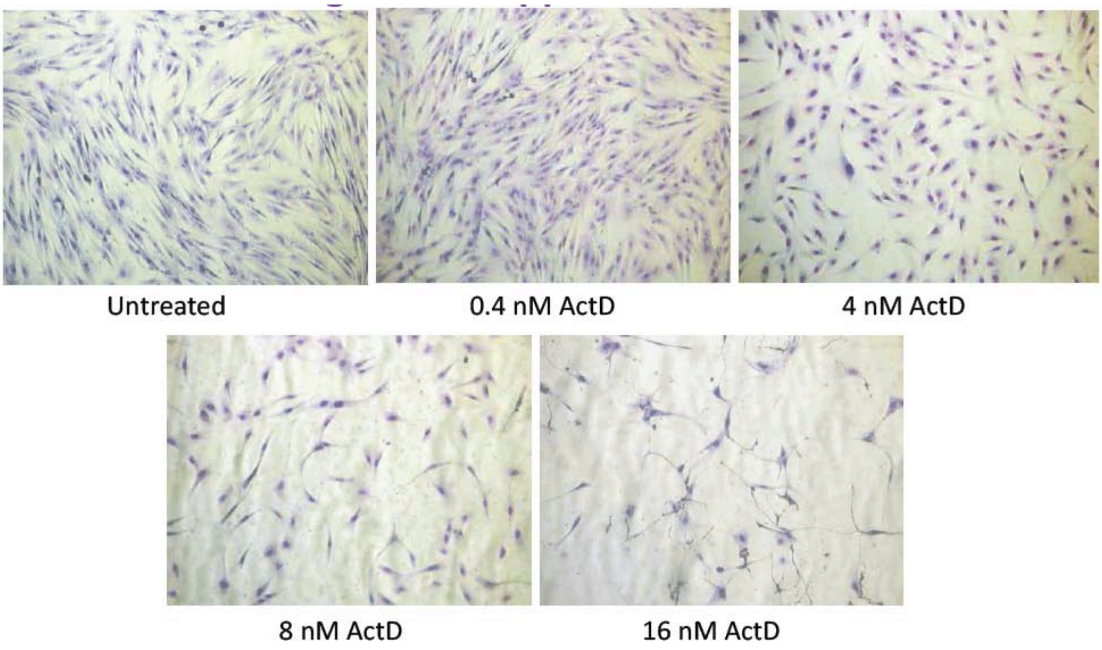

Figure S3B: NDHF cells were treated with the indicated doses of ActD 24 hours post-seeding. The cells were fixed and then stained with Giemsa 24 hours post ActD addition.
